# Medicine

**Published:** 1910-03

**Authors:** Carey Coombs


### Progress of the Medical Sciences.

### MEDICINE.

### Amyotonia Congenita.

In the spring of 1906 a girl suffering from amyotonia congenita was shown at a Bristol meeting of the British Medical Association. This was the first case described as such in England, though the disease was defined by Oppenheim in 1900, and other instances had come under review in Germany, Italy and America. Since that time the literature has been amplified by contributions from these same countries, as well as from England and France, and it is gratifying to find that the fullest and best account of the disease, up to the present time, emanates from Queen's Square. Two papers, the first[1] by J. S. Collier and S. A. K. Wilson, and the second[2] by J. S. Collier and Gordon Holmes, provide us with a knowledge of the disease as exhaustive and accurate as we are likely to possess for many years to come.

The *causation* is obscure. Muscular flaccidity is noted within a few hours of birth as a rule, but in a few instances it seems to have been not congenital but acquired, following acute infections in early infancy. Sex has no determining influence, neither is there a familial factor; the maternal health during pregnancy gives no clue, and, indeed, nothing certain is known as to the origin of the disease, except that as a rule it seems to arise during intra-uterine life.

For our knowledge of the *pathology* we depend upon three autopsies—one recorded by Collier and Holmes,[2] another by Spiller,[3] and a third by Baudouin.[4] Muscle has also been excised during life, and examined by the English observers and by Bing. The muscles are small, and there is a very considerable excess of interstitial fat. Most of the fibres are remarkably slender and loosely packed; among these are a few enormous fibres undergoing regressive changes. The degree of alteration varies widely in the different muscles. The walls of the blood-vessels supplying the muscles are thickened, and in some sections perivascular foci of cellular infiltration are seen. The appearances are therefore similar to those seen in sections of muscle from cases of myopathy. The ventral horns of the cord are poor in cells, and the anterior roots contain relatively few myelinated fibres. Collier and Holmes consider that these data suggest a regressive change rather than a developmental anomaly.

The characteristic *symptoms* are muscular weakness and flaccidity. This latter feature is due to an absolute tonelessness of the affected muscles associated with laxity of ligaments and other structures, so that the limbs can be passively contorted into most curious and gruesome attitudes. The muscular power, though very feeble, is never quite gone, and the muscles feel small and soft, though occasionally contractures are seen. Fibrillary tremor has never been observed. Galvanic currents cause a response, not so Faradic, (in the Bristol case reaction to both was diminished). The distribution of these phenomena is important. It is symmetrical, and may be universal (except that the muscles of chewing and swallowing have been spared in

[1] *Brain*, 1908, xxxi, 1. [2] *Ibid.*, 1909, xxxii, 269.

[3] *Univ. Penna. M. Bull.*, 1905, xvii, 342.

[4] *Semaine Méd.*, 1907, p. 241.

all cases recorded hitherto). The face suffers least often, the lower limbs most often. The muscles are not affected singly, or in groups determined by nerve supply; the affection seizes impartially on nearly all the muscles of a limb, the peripheral muscles always suffering most. Neither mind nor sensation is affected, and the sphincters escape. The deep reflexes are lost in muscles that are profoundly weakened.

The *course* of the disease is towards recovery, provided the period of infancy, with its pulmonary dangers, be overpassed safely. This amelioration is helped by *treatment* consisting of massage, electricity and tonics.

**Syphilis and Aortic Lesions.**

Recent researches have thrown so much light on the causation and diagnosis of syphilis, and so much importance has of late been attached to lesions of the aorta, that it is not surprising to find many data coming to light by which the relation between syphilis and aortic disease may be more precisely defined than heretofore. The evolution of our present knowledge of this matter can be traced from its beginnings in those clinical observations by which aortic insufficiency in middle life, and aortic aneurysm, were linked, in a large percentage of cases, with a history of syphilis. The next step may be said to consist in the separation of mesaortitis from other forms of arterial decrescence, and this particular lesion was soon found by Chiari and others to occur with significant frequency in connection with general paralysis and other syphilitic lesions. To these facts must be added others of more recent discovery. Klotz[1] has noted the occurrence of mesaortitis in infants with inherited syphilis (exemplified by a case of abdominal aneurysm in a young congenital syphilitic, recently shown by Dr. Nixon); Wright,[2] Schmorl,[3] and Benda[4] have all succeeded in demonstrating the spirochæta pallida in connection with the inflammatory foci of mesaortitis; while more recently still Longcope[5] has given a detailed account of twenty-one cases examined *post-mortem*, in which mesaortitis was associated with a chronic deforming inflammation of the aortic valves. In eleven there was a history of syphilis, in three of rheumatic fever; the majority were men in middle life. This group is compared with two others—one of twenty-one cases, in which aortic and mitral endocarditis were associated as a result of rheumatic infection, a group easily separable from the syphilitic cases described above; and another of thirty-four cases, mainly elderly men, with ordinary atheroma of the aorta. What is perhaps of most practical importance is that although in these, as in the cases of mesaortitis, the aortic

[1] *J. Path. and Bacteriol.*, 1907, xii, 11.
[2] *Boston M. and S. J.*, 1909, clx, 539.
*München. med. Wchnschr.*, 1907, liv, 188.
[4] *Berl. klin. Wchnshcr.*, 1906, xliii, 989.
[5] *J. Am. M. Ass.*, January 8th, 1910, liii.

valves were diseased, yet in the syphilitic group signs of aortic insufficiency were nearly always present during life, while in the senile cases such signs were almost never found. The inference to be drawn is that when clinical methods of examination suggest a diagnosis of aortic disease a syphilitic origin may be suspected, especially when there is evidence of aortic without mitral endocarditis. This is confirmed by various observers who have used the Wassermann reaction. Citron[1] found a positive reaction in 62.5 per cent. of a series of cases of aortic regurgitation, without mitral disease; Schulze,[2] in twelve cases of aortic disease, obtained a positive result eleven times; Collins and Sachs[3] in seventeen out of eighteen cases; and Donath[4] in 85 per cent. of his series. To sum up, aortic regurgitation, and other aortic lesions clinically discoverable, are to be regarded as due to syphilis in such cases as are not rheumatic in origin, and should be treated accordingly.

**Sloughing of the Scrotum in the New-born.**

The infective processes of early infancy are characterised by a peculiar intensity and virulence. A very interesting example was described in a paper read several years ago at a Bristol meeting of the British Medical Association, by Dr. Newman Neild, on necrosis of the mammary gland in early infancy, with an associated fatal pyæmia. A similar affection is the subject of a paper by Lilla,[5] who describes the case of a male infant, ill-conditioned as the result of mistakes in feeding, admitted to hospital at the age of two weeks. Six days before a small boil had appeared above the pubes; this was practically cured at the time of admission. The child was feverish and very weak, passing frequent green stools. The scrotum and penis were much swollen; on the latter there were several points of inflammation, and on the scrotum a greyish-white area of necrosis was seen, which on incision proved to implicate the whole thickness of the scrotal wall. Both penis and scrotum were opened up and fomented, and the child recovered; not, however, without incident, for two abscesses appeared, one in the right buttock and the other on the left knee. Lilla refers to about a dozen other similar cases which have been recorded. The principal features are the predisposing influence of malnutrition, the fact that it usually occurs about the end of the first month (which is against the prevailing theory that the portal of infection is the umbilical scar, as it certainly was not in Lilla's own case), its liability to be followed by pyæmia, and the predominance of streptococci as causal agents.

CAREY COOMBS.

[1] *Berl. klin. Wchnschr.*, 1908, xlv, 2,142.
[2] *Deutsche Ztschr. f. Chir.*, 1908, xcv, 1–5.
[3] *Am. J. M. Sc.*, 1909, cxxxviii, 344.
[4] *Berl. klin. Wchnschr.*, November 8th, 1909.
[5] *Gazz. d. Osp. e d. Clin.*, January 6th 1910.

